# Changes in neuronal activation patterns in response to androgen deprivation therapy: a pilot study

**DOI:** 10.1186/1471-2407-10-1

**Published:** 2010-01-04

**Authors:** Monique M Cherrier, Paul R Borghesani, Amy L Shelton, Celestia S Higano

**Affiliations:** 1 Department of Psychiatry and Behavioral Sciences, University of Washington School of Medicine, Seattle, WA; 98195; USA; 2 Department of Medicine, University of Washington School of Medicine, Seattle, WA; 98195, USA; 3 MIRECC VAPSHCS, 1660 S. Columbian Way Seattle WA 98108; USA; 4 Department of Psychological and Brain Sciences, Ames Hall/3400 North Charles Street, Johns Hopkins University, Baltimore, MD 21218: USA

## Abstract

**Background:**

A common treatment option for men with prostate cancer is androgen deprivation therapy (ADT). However, men undergoing ADT may experience physical side effects, changes in quality of life and sometimes psychiatric and cognitive side effects.

**Methods:**

In this study, hormone naïve patients without evidence of metastases with a rising PSA were treated with nine months of ADT. Functional magnetic resonance imaging (fMRI) of the brain during three visuospatial tasks was performed at baseline prior to treatment and after nine months of ADT in five subjects. Seven healthy control patients, underwent neuroimaging at the same time intervals.

**Results:**

ADT patients showed reduced, task-related BOLD-fMRI activation during treatment that was not observed in control subjects. Reduction in activation in right parietal-occipital regions from baseline was observed during recall of the spatial location of objects and mental rotation.

**Conclusions:**

Findings, while preliminary, suggest that ADT reduces task-related neural activation in brain regions that are involved in mental rotation and accurate recall of spatial information.

## Background

Prostate cancer is the most common form of non-skin cancer diagnosed in U.S. men with over 218,000 newly diagnosed cases each year estimated per year [1]. While androgen deprivation therapy (ADT) used to be reserved for those with metastatic disease and resulted in a median survival of 2-5 years [2,3], ADT is now commonly prescribed in the setting of localized disease (e.g. in combination with radiation therapy) or for the 22% of patients who fail primary therapy and have a rising PSA without evidence of metastases [4]. In contrast to those with metastatic disease the latter patients often have no disease related symptoms and may live for many years.

Physiological consequences of androgen deprivation include obesity, anemia, loss of bone mineral density, muscle atrophy, gynecomastia and mood changes [2,5,6]. A recent population based study showed that ADT was associated with an increased risk of diabetes, myocardial infarction, and hypertension [7]. Psychiatric changes can include depression and anxiety [8] and cognitive problems can include impairments in verbal memory [9,10], spatial reasoning [11] and attention [12]. Given these cognitive and medical side effects of ADT understanding the immediate and long-term consequence of treatment is essential.

Based on findings from our previous study demonstrating a decline in spatial reasoning abilities in healthy men undergoing ADT [11], the aim of this study was to examine if neural activation changes were evident in response to androgen deprivation while performing spatial reasoning and spatial memory tasks using functional magnetic resonance imaging (fMRI). Spatial reasoning and spatial memory were specifically chosen to examine the effects of ADT on brain function because task-related brain activation has been clearly identified, particularly in regions of the parietal cortex [13-16] along with evidence of hormone influences on these brain regions [17-19]. The aim of the present study was to assess changes in neural activation in response to androgen deprivation using functional magnetic resonance imaging (fMRI). We hypothesized that men undergoing ADT would show reduced activation in the task-related parietal region during spatial reasoning and spatial memory tasks compared to baseline and to healthy controls.

## Methods

### Participants

Participants were a subset of participants from a larger study examining cognitive and mood changes in ADT who agreed to the additional neuroimaging procedures (See [20] for details). The sample is a convenience sample comprised of those participants who were willing to undergo neuroimaging procedures in addition to the cognitive assessment procedures in the larger study. Twelve prostate cancer patients with rising PSA (biochemical relapse) following primary therapy (radiation, brachytherapy or prostatectomy) in which intermittent androgen suppression (ADT) therapy is indicated were eligible and gave informed consent to participate. Intermittent ADT is a treatment strategy that cycles androgen withdrawal with an "off treatment" period allowing the testosterone levels to return to eugonadal levels. This approach has allowed for characterization of the biological, psychological, and quality of life effects of ADT and which effects are reversible or attenuated when the testosterone levels increase during the 'off treatment' period. Additional eligibility criteria included age greater than 21 years, no disturbance in daily functioning and normal pretreatment serum testosterone. Exclusion criteria included moderate pain related to prostate cancer, history of prior psychiatric illness involving hospitalization, dementia, central nervous system metastasis, history of systemic chemotherapy, current renal dysfunction or hepatic dysfunction, prior exposure to welding or any surgery resulting in metal implants in the brain or presence of any unusual findings from magnetic resonance imaging (MRI). The University of Washington Institutional Review Board approved this study and written informed consent was obtained from all patients prior to study procedures. A placebo or non-treated group in this population was not feasible, therefore a comparable non-treatment group of twelve community dwelling eugonadal men without prostate cancer range matched for age within four years and education within two years underwent the same neuroimaging procedures at a matching time interval to the ADT group. Radiology technicians who performed scanning operations were blind to conditions. Imaging data were identified only by coded numbers and group membership was coded during image analysis.

### Study Design

Baseline evaluations for ADT patients included a bone scan, CT scan of the abdomen and pelvis, chest x-ray, cognitive function testing, and baseline laboratory values including renal and hepatic function, PSA and total testosterone. Patients were treated with an anti-androgen (flutamide 250 mg TID or bicalutamide 50 mg per day if intolerant of flutamide) lead-in alone for 2 weeks followed by the addition of leuprolide 7.5 mg administered by monthly intramuscular injection for nine doses. Thereafter, the PSA was followed monthly. The ADT group underwent neuroimaging procedures prior to the start of treatment (time_1_) and again after nine months of androgen deprivation (time_2_). The control group underwent two neuroimaging sessions nine months apart. In addition to high resolution structural scans, subjects underwent blood oxygen level dependent (BOLD) -fMRI imaging while performing three cognitive tasks; i) encoding and ii) recognition in a spatial memory task and iii) a mental rotation task.

### Environmental Memory Task (EMT)

An environmental memory task designed by Shelton and colleagues [21,22] was used for a spatial memory task. The task was modified for older individuals by using a single environment (either a park or market) during both encoding and recognition portions and allowing subjects 6 seconds (rather then 3) to identify still images during recognition. This task is composed of separate, sequentially presented encoding and recognition runs lasting 348 and 478 seconds, respectively.

During both encoding and recognition the environment is viewed from the ground (route) and the aerial (survey) perspective in an alternating, pseudorandom order. To ensure that subjects were familiar with identifying objects from the route and survey perspective and that they understood the requirements of the task they completed a practice session outside the MRI suite with an environment not subsequently used during the scanning session. All subjects demonstrated satisfactory understanding and performance of the task after this practice session and the practice session was repeated at the second visit. During the fMRI session verbal instructions were provided though headphones and visual stimuli through goggles connected via high-resolution fiber optic cables. Stimuli were presented using an Apple computer running PsyScope [23]. Radiology technicians who were responsible for scanning the participants were blind to condition and only subject numbers identified the imaging data.

### Encoding task (ENCODE)

During the encoding task subjects viewed animated movies of an environment (either a park or market) presented from both the route perspective and the survey perspective. The environment was shown six times, 3× from the route and 3× from the survey perspective, in an alternating psuedorandom order. The individual movies were 46 seconds long and were preceded by 3 seconds of fixation. Three, 18 second fixation blocks (cross-hair) were intermixed in the sequence. The run was thus composed of 3 fixation blocks (18s each) + 3 repetitions of the route perspective movie (46+3s each) + 3 repetitions of the survey perspective movie (46+3s each) in for a total run time or 348 s (116 volumes, TR = 3). Immediately following encoding subjects were tested on their knowledge of the complex environment during the recognition task (see below). Subjects were informed that their memory for the environment would be subsequently tested but active responses were not required during encoding.

### Recognition task (RECOG)

During the recognition run subjects viewed still images of the previously studied environment and determined if objects within the environment were; (i) correctly positioned (previously seen items in appropriate environmental locations), (ii) spatially rearranged (previously seen items shuffled within the environment), or (iii) they could not tell (do not know). A total of 64 images were presented; 32 from the route perspective (4 blocks of 8 images, each block preceded by 3 s of fixation) and 32 images from the survey perspective (4 blocks of 8 images, each block preceded by 3 s of fixation). Each set of 8 images contained four spatially correct images and four foils or incorrect choices with the order randomized. Four, 18 s fixation blocks (cross-hair) were intermixed in the sequence. The entire run was thus composed of 4 fixation blocks (18s each) + 4 route perspective picture blocks (48+3s each) + 4 survey perspective picture blocks (48+3s each) in a quasi-random order for a total run time of 480s (160 volumes, TR = 3). Following the fMRI session subjects were asked to draw the environment to ensure they had completed the task appropriately (data not scored).

### Mental Rotation Matching Task (MATCH)

Participants were shown black and white complex, three dimensional (3D) figures taken from the Shephard and Metzler Mental Rotation test [24,25] or complex and abstract two dimensional (2D) figures in black/white/gray. Participants were asked to determine if the figures (i) matched exactly or, (ii) they did not match or, (iii) did not know. Incorrect matches for the three-dimension figures were either mirror images or a different configuration whereas a match was the same image side by side, although the images may have been rotated in space. For the 2D- images, a match was the same image side by side, whereas a mis-match involved one of the figures having additional details or missing details. Stimuli were presented using Eprime^® ^software http://www.pstnet.com/products/e-prime/ and responses were recorded using magnet compatible buttons. For 3D-figures a total of 15 figures were presented (3 blocks of 5 images, each block preceded by 6 seconds of instruction) and for 2D-figures a total of 18 images were presented (3 blocks of 10 images, each block preceded by 6 seconds of instruction). Four, 18 s fixation blocks (cross-hair) were inter-mixed in the sequence. The entire run was thus composed of 4 fixation blocks (18 s each) + 6 figure matching blocks + 6 instruction periods for a total run time of 468 second scan (156 volumes, TR = 3).

### BOLD imaging

Structural and functional MRI were performed on a 1.5 T MR imaging system (General Electric, Waukesha, WI). Whole brain axial anatomic (21 images, TR/TE 200/2.2 ms) and functional (21 images, TR/TE 3000/50 ms, flip angle 90°, 64 × 64 matrix, in-plane resolution of 3.75 × 3.75 mm, slice thickness 6 mm with 1 mm spaces) images were collected using a two-dimensional gradient-echo, echo-planar pulse sequence.

### Functional image analysis

Functional data were processed using tools from the FMRIB Software Library (http://www.fmrib.ox.ac.uk and Smith et al., 2004) in essentially 6 steps (see Table 1): STEP 1) Independent components analysis based artifact detection and component removal, STEP 2) first-level analysis of individual paradigms for each subject; STEP 3) second-level fixed-effects analysis of individual subject's time_1 _vs. time_2 _scans; STEP 4) group mixed-effects general linear model comparison of ADT treated and CTR groups and, STEP 5) group mixed-effects comparison of baseline (time_1_) vs. treatment (time_2_) activation changes in each group for the individual tasks; STEP 6) Using paradigm specific ROIs defined by STEP 5 (i.e., a unique ROI for ENCODE, RECOG and MATCH) the average change in the z-score between time_1 _and time_2 _was determined for each subject and task.

**Table 1:** SUMMARY OF STATISTICAL COMPARISONS

	Inputs	EVs in model	Output/contrasts
**STEP 1**:	ENCODE data	n.a.	ENCODE* data
ICA data review (Separate for each paradigm and subject)	RECOG data		RECOG* data
	MATCH data		MATCH* data
**STEP 2**:	ENCODE* data	EV1: route (or 3D)	C_1_1 = EV1-EV2
Subject paradigm analysis (Separate for each paradigm and subject)	RECOG* data	EV2: survey (or 2D)	C_1_2 = EV2-EV1
	MATCH* data	EV3: fixation	C_1_3 = (EV1+EV2)-2*EV3
		EV4-9: motion parameters of no interest	C_1_4 = 2*EV3-(EV1+EV2)
**STEP 3**:	C_1_3 from each paradigm	EV1: time_1 _> time_2_	C_2_1 = EV1
Intra-subject analysis (Separate for each subject)		EV2-4: paired t-test parameters of no interest	C_2_2 = -EV1
**STEP 4**:	C_2_1 from each subject	EV1: ADT	C_3_1 = EV1
Group time_1 _vs. time_2 _analysis		EV2: CTR	C_3_2 = -EV1
			C_3_3 = EV2
			C_3_4 = -EV2
			C_3_5 = EV1-EV2
			C_3_6 = EV2-EV1
**STEP 5**:	C_1_1 or C_1_3 from each subject	EV1: ADT_time1 _> ADT_time2_	C_4_1 = EV1
Group paradigm analysis (Separate for each paradigm)		EV2: CTR_time1 _> CTR_time2_	C_4_2 = -EV1
		EV3-14: paired t-test	C_4_3 = EV2
		parameters of no interest	C_4_4 = -EV2
			C_4_5 = EV1-EV2
			C_4_6 = EV2-EV1
**STEP 6**:	C_1_3 from each subject	n.a.	n.a.
Determining average z-scores (Separate for each paradigm and subject)			

Independent components analysis (ICA), explanatory variable (EV), contrast (C)

STEP 1: Motion correction [26], spatial smoothing with a 7 mm at half-maximum Gaussian kernel and low pass (sigma = 2.8 s) and high pass (sigma = 75 s) filters were applied. Absolute motion varied from 0.1 to 1.2 mm and relative motion varied between 0.04 and 0.22 mm. Data were then analyzed using probabilististic independent components analysis (ICA) (MELODIC) [27] to identify extreme scanning artifacts and the corresponding components were removed. 4-10 components removed from 4 of 24 ENCODE scans (1 ADT and 1 CTR subject) and 2 or 24 MATCH scans (1 CTR subject). No RECOG scan needed correction. STEP 2: The initial 6 volumes of each functional series were discarded to allow T1 equilibrium. For each task three separate explanatory variables (EVs) plus 6 motion parameters of no interest were modeled. For each subject and each task, four individual contrasts were designed to determine task-related activation, deactivation, and the difference between route/survey in the EMT and 3D/2D in the MATCH tasks. STEP 3: Each subject's functional data was mapped onto their high-resolution structural images using 7 DOF and into standard space (MNI152 brain) with a 12 DOF affine transformation [28]. Registration was confirmed by visual inspection and manually re-aligned as appropriate. Individually for each subject, task-related activation at time_1 _was compared to time_2 _using a fixed-effects paired t-test. This allowed us to assess the change in task-related activation averaged across the three tasks for each subject. STEP 4: Between-group comparisons were done using a full mixed-effects GLM using the parameter estimates from STEP 3 as inputs [29,30] and activated clusters were identified based on random field theory correcting for multiple comparisons (FLAME). STEP 5: A post-hoc region of interest (ROI) analysis was performed separately for each paradigm to determine how time_1 _vs. time_2 _changes in the individual paradigm contributed to the findings in STEP 4. For STEP 4 a z-threshold of 2.7 and p < 0.01 was set to minimize type 1 error while for STEP 5 stringency was relaxed by decreasing the z-threshold to 2.0 and p < 0.05 to reduce type 2 error. STEP 6: A unique statistically defined ROI was generated for each paradigm corresponding to the region where ΔADT > ΔCTR (RECOG and MATCH) or ADT_time1 _> ADT_time2 _∩ CTR_time1 _> CTR_time2 _(ENCODE). These three paradigm specific ROIs were used in the calculation of the average z-scores for individual subjects during the corresponding tasks within the parietal lobe.

## Results

Both ADT patients and controls (CTR) ranged in age from 56 to 78 years with a mean age of 65 years and education ranged from 12 to 21 years with a mean education level of 17 years. Because the groups were range matched on age and education they were not significantly different on these demographic variables. One CTR and one ADT participant dropped out prior to baseline scanning due to concerns about 'feeling closed in' in the scanner. One CTR participant and two ADT patients did not complete the month 9 imaging session due to scheduling conflicts. Three CTR and ADT patients were not analyzed due to excessive motion. In all, seven controls and five ADT patients were included in the imaging analysis. All participants were right handed. Each subject was scanned twice (baseline, 9 months) with 24 scans sessions and three tasks for a total of 72 runs included in the analysis. Similar to other functional neuroimaging studies, variance in the BOLD signal for our study, for most brain regions across all paradigms was about 1% of the total signal. Given the design of our paradigms this would allow us to detect a 0.5% increase in signal (task-associated activation) with about 50% power and 0.75% change with > 80%power in individual subjects. Review of clusters of significant task-associated activation within individual subjects revealed, as expected from the simplistic prior power calculation, signal changes ranged 0.25-1.0% of the signal (data not shown)

As expected, total testosterone was reduced significantly in the treatment group at the time of the second scan compared to the control group F(1,9) = 37.6, p < .01 and a significant group by time interaction F (1,9) 53.26, p < .01 was evident. The ADT group total serum testosterone levels declined from an average of 3.52 (0.33) ng/ml at baseline to 0.25 (0.10) at month 9. The control group did not change with a baseline testosterone of 3.8 (0.67) ng/ml and a month 9 average of 4.3 (1.3) ng/ml.

In scanner behavioral performance on the environmental memory task (EMT) or mental rotation did not change significantly for either group, 36.0 (10) average total correct EMT (64 points possible) at time_1 _and 35.75 (13.6) at time_2 _for ADT group and 31.3 (13.5) at time_1 _and 38.0 (10.9) at time_2 _for control group.

**Figure 1:**
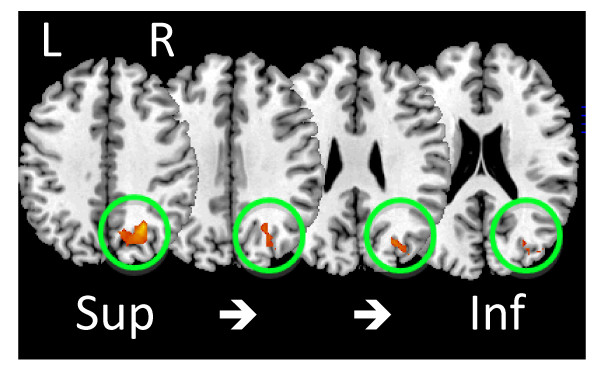
**Reduced left parietal activation in the ADT group at time_2_**. Four sections of a standardized brain with the area of reduced activation in the ADT group are shown (outlined by the green circle). No other areas of reduced activation were noted. See Methods and Table 2 for statistical details, *p *= 0.01 for cluster depicted.

**Figure 2:**
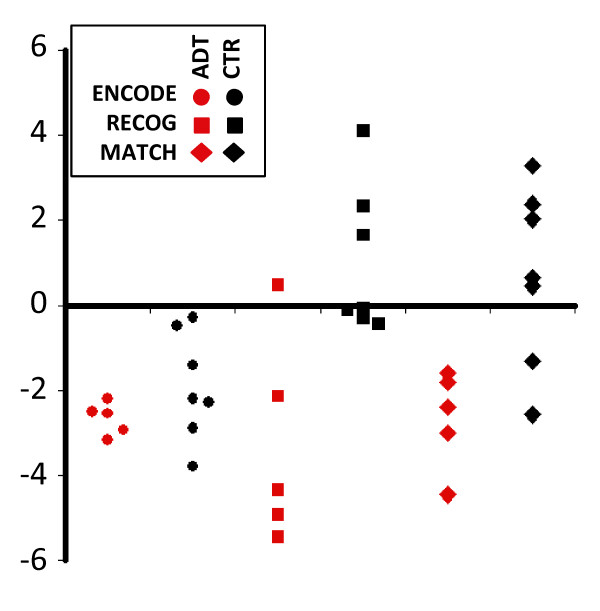
**Reduced task-related activation in individual subjects during encoding, recognition and rotation at time_2_**. Task specific ROIs were defined by significant activation (over crosshair viewing) during the individual tasks in a mixed-effects GLM and the average Z-scores within these regions were determined for each subject (and for each task) at time_1 _and time_2_. For each subject, the difference in average z-scores was calculated (time_2 _- time_1_) and thus negative scores represent a decrease in task-related activation within an individual. Each point represents time_2 _- time_1 _from a single subject. See Table 2 for statistical details.

To determine whether ADT affected neurovascular responses during these tasks, BOLD-fMRI was acquired prior to treatment (time1) and after 9-months of ADT (or no treatment in the control group) (time2) in both the ADT and CTR groups. Task-related activation, defined as the difference in the BOLD signal between the active tasks (which differ between paradigms) and viewing crosshairs (fixation), was reduced in a single right parietal region in the ADT group but not the CTR group (Figure 1). This region extended superiorly from the right precuneal area and inferiorly to the lateral occipital lobe. BOLD responses during individual paradigms were differentially affected (Table 2 and Figure 2). Task-related activation in this region was reduced in both the ADT and CTR groups during ENCODE. In contrast, during RECOG and MATCH, task-related activation was reduced within the ADT group but not the CTR group and was significantly different between groups. To ensure these findings were not the result of an activation difference between groups at time1 an intergroup analysis was performed for each paradigm. No activation differences at time1 were found between groups.

**Table 2:** ACTIVATION CHANGES IN THE RIGHT PARIETAL AREA BETWEEN BASELINE (TIME 1) & TREATMENT (TIME 2)

Paradigm	Contrast	Brain region of cluster (all are right sided)	Cluster p-value	Area in cm3	Z-max	Z-max MNI coordinates
All paradigms	ADT_Time1 _> ADT_time2_	precuneus, cuneus and lateral occipital lobe	0.011	4.72	4.15	26,-62,40
ENCODE	ADT_time1 _> ADT_time2_	cuneus and lateral occipital lobe	0.006	3.9	4.37	22,-88,-4
		precuneus	0.028	2.15	4.71	26,-68,44
	CTR_time1 _> CTR_time2_	precuneus, cuneus and lateral occipital lobe	0.002	5.63	5.14	18,-72,46
RECOG	ADT_time1 _> ADT_time2_	cuneus and precuneus	0.001	6.74	4.88	26,-64,36
	ΔADT > ΔCTR	precuneus	0.032	2.17	3.27	26,-64,36
MATCH	ADT_time1 _> ADT_time2_	cuneus and precuneus	0.002	6.26	5.67	34,-90,8
	ΔADT > ΔCTR	precuneus	0.031	2.34	3.65	34,-82,32

## Discussion

Our results indicate androgen deprivation therapy (ADT) patients had reduced task-related BOLD-fMRI activation following androgen deprivation in the right parietal-occipital region for tasks involving manipulation and recall of spatial information. Although in scanner performance did not decline for either task in this small sample the observed reduced task-related activation may help explain our previous findings of impaired rotational abilities in a larger sample of men undergoing ADT [11,20]. Cognitive changes in men undergoing ADT using paper and pencil and computerized tests have found impairments in verbal memory [9,10], spatial abilities [11,20] and attention [12]. However, declines may be evident for only a subset of participants [31], and some studies have found no appreciable change in cognition from androgen ablation therapy [32]. These findings suggest that gonadal steroids modulate parietal-occipital function and are consistent with a report correlating endogenous testosterone levels and neural activation in the inferior parietal lobule and left supramarginal gyrus regions while performing a mental rotation task [18].

Neuroimaging studies of mental rotation have generally found that rotation of three dimensional figures engages occipital and parietal regions with numerous studies supporting the role of the superior parietal lobule in the performance of mental rotation [33,34]. Gender differences have been reported suggesting that women engage or recruit additional brain regions such as the frontal lobes, however the parietal region appears to be reliably activated by both genders [17,35,36]. Several studies but not all [17] have suggested that men outperform women on the mental rotation task [37] with some evidence for a positive relationship between performance on the task and circulating T levels [38,39]. Our results are certainly consistent with these previous studies in demonstrating that parietal regions are activated during the mental rotation task and extend these findings to reveal potential interactions with hormone levels.

In addition to changes observed in the object rotation task, we also observed changes in neural activation from ADT during the recognition portion of the environmental memory task. Encoding of the complex environments have been reliably shown to activate fusiform gyrus, anterior and posterior superior parietal cortex, medial frontal cortex, and occipital cortex [21,40] similar to patterns of activation observed for the present study. However, only the ADT group demonstrated a decrease in task-related activity at month 9 of androgen deprivation treatment compared to baseline in the right parietal lobe during the recognition portion of the EMT while both groups had decreased task-related activation during encoding. Both the environmental memory task and the mental rotation matching task have been shown to activate the parietal region and there is some suggestion that this region may be related to spatial transformation processes (See [40] for discussion).

Studies utilizing positron emission tomography (PET) techniques have also reported changes in neural activation in response to changes in androgen levels. Hypogonadal (low testosterone) men demonstrate increases in cerebral glucose metabolism after 12 weeks of testosterone treatment compared in occipital and frontal regions while performing a mental rotation task as measured by F-18-deoxyglucose (FDG) PET [41]. A longitudinal study of healthy, eugonadal older men found that higher free testosterone (T) levels were related with increased regional cerebral blood flow (rCBF) as measured by serial [^15^O] PET in the hippocampus bilaterally, anterior cingulate gyrus and right inferior frontal cortex during resting state [42]. A recent study of testosterone supplementation in eugonadal men reported both increases and decreases in regional cerebral blood flow (rCBF) as measured by [F-18] fluorodeoxy glucose (FDG) while performing a verbal memory recognition task [43]. Testosterone treatment resulted in increased glucose utilization in left entorhinal cortex, right posterior hippocampus and right parahippocampal gyrus areas and decreased glucose utilization in right entorhinal cortex/amygdala. A single summary of findings from these studies is difficult given the wide variation of tasks from spatial to verbal and resting state as well as populations ranging from eugonadal and hypogonadal. However, there does appear to be general support for a modulation of neural activity in response to manipulation of androgens. A consistent pattern of findings amongst studies likely awaits further replication of hormone manipulation, imaging technique and cognitive task.

Results of the current study suggest that circulating androgen levels may modulate neural activity, particularly with regard to tasks that are spatially demanding or require spatial transformation processing. In particular, declines in neural activity were evident only in the ADT group compared to baseline and were most pronounced for those tasks with spatial demand such as three-dimensional rotation and the recall of the spatial location of objects from the EMT. The clinical relevance of these tasks relates to everyday tasks such as constructing or putting together furniture, household items or building structures as well as map reading and navigating which often require spatial or three dimensional mental transformations. Although we have demonstrated objective evidence of declines in spatial abilities in men undergoing ADT [11,20], ideally these should correspond to subjective reports of everyday task difficulties. We are currently, investigating the nature of subjective cognitive complaints in ADT patients. However, it is not unusual for patients to demonstrate poor self or subjective appraisal of cognitive abilities such that objective and subjective measurement do not match (see for example [44-46]). This has also been observed in cancer patients (e.g. Jacobs (2007) [47]) who may be undergoing tremendous personal and health challenges. The neuroimaging results provide further convergence of evidence and a neural link with regard to our earlier observed findings of declines in spatial abilities.

While these results are interesting the sample size was limited. Thus, results will need to be independently replicated with a larger sample. Nonetheless, the same decline in task related activation following ADT was evident for both cognitive tasks thus lending some support to consistency despite the small sample size along with the study design which was prospective and repeated measure thus there were two scans analyzed for each subject. In addition, the study was prospective and enrolled participants prior to the start of treatment, thus establishing a clean baseline and the sample size is consistent with other studies involving cancer patients and neuroimaging (See McDonald et al. (2008) [48] for a review) or studies involving coordination of neuroimaging along with treatment. An additional consideration is that the sample was highly educated (mean of 17 years). This may have had an impact on the participants' ability to perform the cognitive tasks and may also provide considerations for the generalizabiltiy of the results to the population of men with prostate cancer. However, patients were range matched to controls for age and education, which may have provided some control over differences between the groups.

## Competing interests

The authors declare that they have no competing interests.

## Authors' contributions

MC participated in the design & coordination of the study, collection of data, data analysis and manuscript preparation. PB participated in the data analysis and manuscript preparation. AS participated in the design of a fMRI cognitive task and data analysis and manuscript preparation. CSH participated in the design and coordination of the study, data collection and manuscript preparation.

## Pre-publication history

The pre-publication history for this paper can be accessed here:

http://www.biomedcentral.com/1471-2407/10/1/prepub
